# Rapid magma ascent beneath La Palma revealed by seismic tomography

**DOI:** 10.1038/s41598-022-21818-9

**Published:** 2022-10-21

**Authors:** Luca D’Auria, Ivan Koulakov, Janire Prudencio, Iván Cabrera-Pérez, Jesús M. Ibáñez, Jose Barrancos, Rubén García-Hernández, David Martínez van Dorth, Germán D. Padilla, Monika Przeor, Victor Ortega, Pedro Hernández, Nemesio M. Peréz

**Affiliations:** 1 Instituto Volcanológico de Canarias (INVOLCAN), Calle Álvaro Martín Díaz, 2, San Cristóbal de La Laguna, Tenerife Spain; 2 Instituto Tecnológico Y de Energías Renovables (ITER), Polígono Industrial de Granadilla s/n, 38600 Granadilla de Abona, Tenerife Spain; 3 Trofimuk Institute of Petroleum Geology and Geophysics SB RAS, Prospekt Koptyuga, 3, 630090 Novosibirsk, Russia; 4 Institute of the Earth’s Crust SB RAS, Lermontova 128, Irkutsk, Russia; 5 Department of Theoretical Physics and Cosmos, Science Faculty, University of Granada, Avd. Fuenteneueva s/n, 18071 Granada, Spain; 6 Andalusian Institute of Geophysiscs, Campus de Cartuja, University of Granada, C/Profesor Clavera 12, 18071 Granada, Spain

**Keywords:** Solid Earth sciences, Geophysics, Seismology, Volcanology

## Abstract

For the first time, we obtained high-resolution images of Earth's interior of the La Palma volcanic eruption that occurred in 2021 derived during the eruptive process. We present evidence of a rapid magmatic rise from the base of the oceanic crust under the island to produce an eruption that was active for 85 days. This eruption is interpreted as a very accelerated and energetic process. We used data from 11,349 earthquakes to perform travel-time seismic tomography. We present high-precision earthquake relocations and 3D distributions of P and S-wave velocities highlighting the geometry of magma sources. We identified three distinct structures: (1) a shallow localised region (< 3 km) of hydrothermal alteration; (2) spatially extensive, consolidated, oceanic crust extending to 10 km depth and; (3) a large sub-crustal magma-filled rock volume intrusion extending from 7 to 25 km depth. Our results suggest that this large magma reservoir feeds the La Palma eruption continuously. Prior to eruption onset, magma ascended from 10 km depth to the surface in less than 7 days. In the upper 3 km, melt migration is along the western contact between consolidated oceanic crust and altered hydrothermal material.

## Introduction

**Figure 1:**
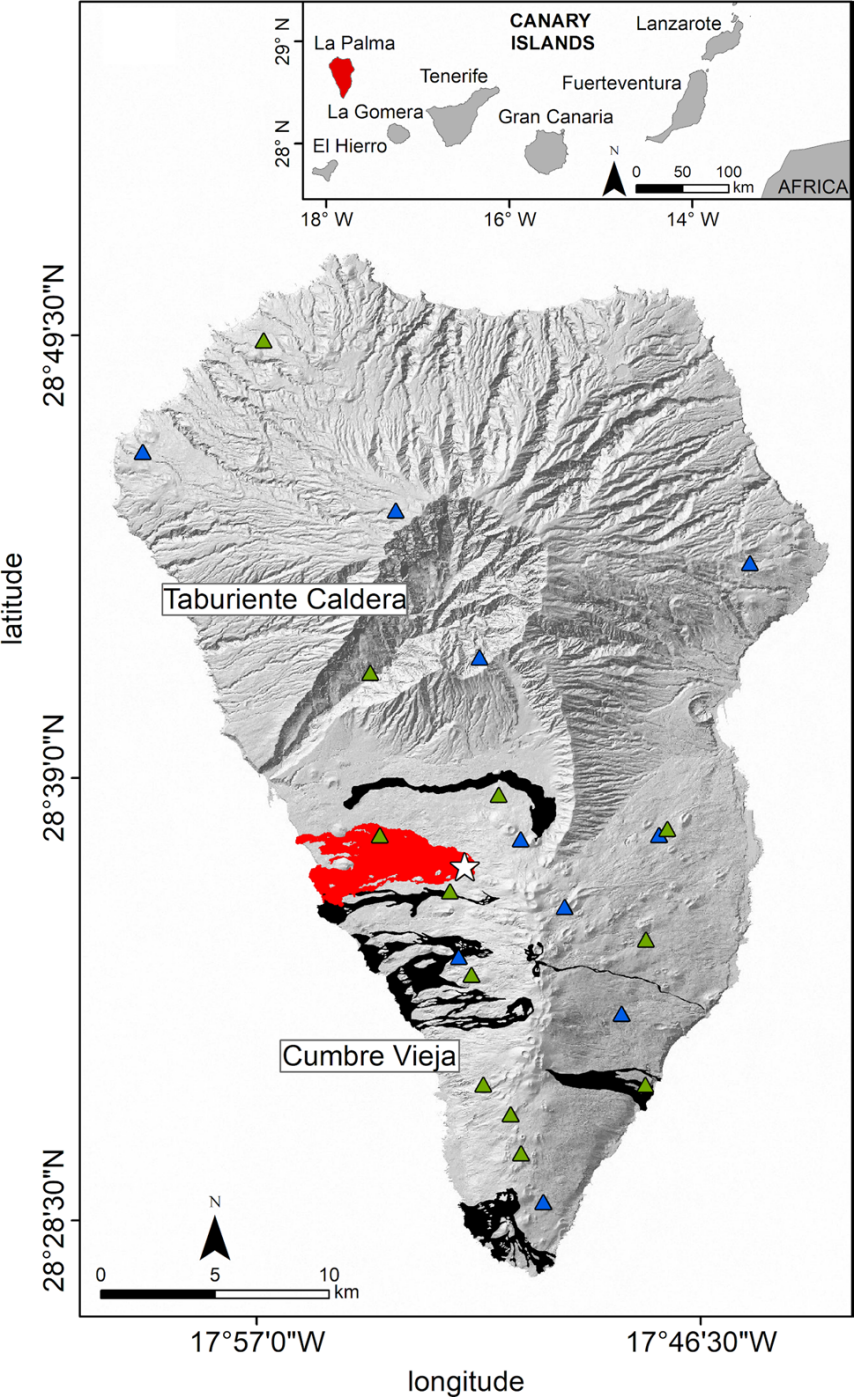
Digital elevation map of La Palma Island with the locations of seismic networks INVOLCAN (green triangles) and IGN (blue triangles). Black shading denotes lava flows from historical eruptions and red shading denotes lava flows from the 2021 activity. The white star is the vent of the 2021 eruption. The digital elevation model and historical lava flows were downloaded from the public graphic repository of GrafCan (www.grafcan.es). The 2021 lava flow was downloaded from the European agency Copernicus Emergency Management Service (httts://emergency.copernicus.eu/mapping/list-of-components/EMSR546). The software used to generate this figure was QGIS 3.22 (https://www.qgis.org).

On 19 September 2021, an eruption of high social and scientific impact began on the island of La Palma, Canary Islands, Spain (Fig. 1) and lasted 85 days until 13 December 2021. This eruption did not cause any casualties, but it destroyed hundreds of homes, disrupted transport and communication networks, and affected extensive areas of farmland that are key to the local economy. In less than three months, this fissure eruption formed a lava field of > 12 km^2^ with thicknesses of up to tens of meters at some points^1^. Among seven previous eruptions on La Palma in the last 500 years^2^, the most productive one formed a lava field of just 4.4 km^2^ (Fig. 1). The eruptive style has been mainly effusive, but with numerous Strombolian explosions, extensive ash fall, and repeated partial collapses of the cone^3^. Intense seismicity activity before and during the eruption has included earthquakes with high magnitudes for this volcano type (up to Ml = 4.3).

**Figure 2:**
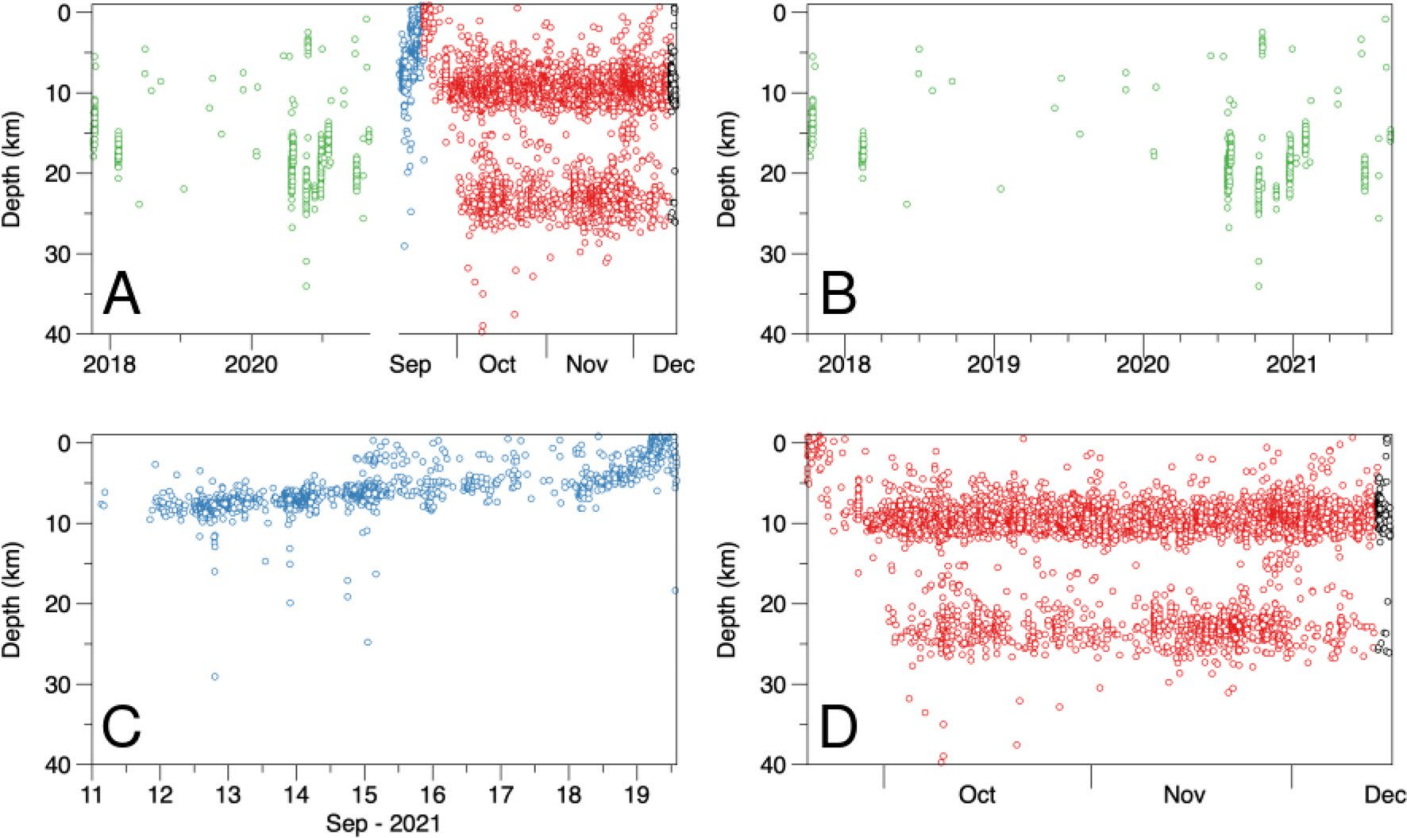
(**A**) Temporal and depth distribution of the used seismicity beneath La Palma (**A**) Seismicity in the entire observation time divided in four periods depicted with different colours. (**B**) Precursory period, from October 2017 to August 2021, plotted in green and zoomed in (**B**). (**C**) Pre-eruptive period in, the week before the start of the eruption. , plotted in blue and zoomed in (**C**). (**D**) Syn-Eruptive and post-eruptive, the seismicity occurred after the start of the eruption up to December 13th 2021, plotted in red, and the post-eruptive, the seismicity after volcanic activity ceased, December 13–16, 2021, plotted in black, all of them represented in (**D**).

Volcano seismology remains one of the most important tools for volcano monitoring. As one of its most significant developments, seismic tomography provides a window into sub-surface structures and their links to magmatic processes^4,5^. However, obtaining tomographic images during eruptions is complex owing to the requirement of high numbers of seismic stations and earthquakes with adequate spatio-temporal distributions to provide sufficient resolution. In most cases, due to logistical problems, the deployment of arrays with a sufficient number of stations for high-quality tomography study during the most active stage of an eruption is difficult and especially rare when such a dense network operated prior to an eruption culmination. Fortunately, all stages of the La Palma eruption were recorded by two high-quality networks that gave us a unique chance to observe details of the spatial-temporary evolution of seismicity before, during and after the most active stage of the eruption (Figs. 2, and S1–S3 in Supplementary). Furthermore, it provided sufficient material to implement high-quality tomography inversion and to infer the 3D structure of the magma plumbing system *during* the active stage of the eruption. Our results have great scientific and social impact and offer improved understanding of pre- and syn-eruptive activity, along with the possible future volcanic scenarios in La Palma Island.

### Previous studies of La Palma and surrounding islands

There is a lack of consensus on the origin and evolution of the Canary Islands archipelago, in part because knowledge of deep structures is scarce and inconclusive. One of the models supports the existence of a mantle plume beneath the western Canary Islands (El Hierro and La Palma)^6,7^. In this model, material flows from the plume to the east, crossing the north-western African continent, travelling along the base of the oceanic lithosphere below the Canary Islands, and finally flowing into a sub-continental lithospheric corridor beneath the Atlas Mountain system.

Using compressional to shear (P to S) converted seismic phases from teleseismic receiver functions, Lodge et al.^8^ studied the crustal and upper mantle structure below La Palma. They identified discontinuities at ∼8 and 14 km depth, and interpreted the deeper one as Moho. However, it cannot be excluded that the discontinuity at ~ 8 km depth may correspond to the Moho, while that at 14 km might be the trace of magmatic underplating.

Based on P-receiver functions, Martínez-Arévalo et al.^9^ suggested that the Moho discontinuity beneath the Canary Islands deepens towards the east, varying in depth from 11.5 to 12.5 km beneath the western islands (El Hierro and La Palma) and up to 20–30 km beneath the eastern islands (Lanzarote and Fuerteventura). They identified a low velocity layer beneath the lithospheric mantle (at 45–65 km depth), which they interpret as a large plume feeding the Canary Islands volcanic system.

From seismic velocity tomography of El Hierro Island, García-Yeguas et al.^10^ determined the base of the oceanic crust to be at 10–12 km depth. They did not observe a magma reservoir. Based on their results, they suggest that the magma intrusion responsible of the 2011 eruptive process warped the Moho below the island, causing localised thinning of the oceanic crust to < 8 km.

Numerous seismic tomography studies of volcanic regions have identified shallow low-velocity structures that are interpreted as highly fractured, unconsolidated, and/or hydrothermally altered volcanic materials, including those for Deception Island volcano^11–13^ , Avacha group volcanoes^14^ and Mt. Etna^15,16^. Similar anomalies have been observed beneath the island of Tenerife^17,18^.

Most information on the internal structure of La Palma was derived based on the magnetotelluric sounding^19^. The obtained 3D geoelectric model demonstrated high-resistivity zone beneath the Taburiente Caldera, which was interpreted as a trace of old highly consolidated intrusive body. At the same time, this model revealed contrasted low-resistivity anomalies around the Cumbre Vieja that may be associated with the presence of high-fractured and/or hydrothermally altered rocks.

On La Palma, no seismic tomography models nor detailed structural geophysical models were previously constructed. In this study, we present the first seismic velocity model for this area.

### Data and algorithms

In this study, we merged datasets derived by the two governmental agencies operating on La Palma prior to and during the eruption: the Instituto Geográfico Nacional (IGN; 11 stations) and the Instituto Volcanológico de Canarias (INVOLCAN; 12 stations) (Fig. 1). Both networks consist of three-component broadband seismic stations sampled with a rate of 100 Hz. The dataset covers the time span from 8 October 2017 to 15 December 2021. The seismic activity (Fig. 2) begins with a seismic swarm at the end of the year 2017. This seismicity, related to the magmatic reactivation process, shows two different space–time characteristics. In the period from 8 October 2017 to August 2021 (Figure S1), seismic activity is recorded mainly at depths between 15 and 20 km, and which in this work we will call precursory activity. On the other hand, we used the term pre-eruptive for the seismic activity associated with the week prior to the eruption (Fig. S2), where a clear migration of seismicity towards the surface is observed, marking the final path followed by the magma until the eruption. Finally, the seismic activity that accompanies the eruptive process was called syn-eruptive seismicity (Fig. S3).

When processing the data, these two agencies used different source location algorithms and different velocity models. Therefore, they provided slightly different solutions for the coordinates and origin times of the same events. When merging, we assumed that both agencies had recorded the same event if the difference between the origin times in the provided solutions was < 1 s. We analysed different values of this threshold and found that when using a smaller value (0.5 s), the number of the common events were much smaller. On the other hand, when using a larger threshold of 2 s, the number of the common events did not increase significantly. From this analysis, we concluded that 1 s is an appropriate value suitable for this case. The merged dataset contains 13,681 events recorded by 23 stations, with 140,078 *P* wave and 155,231 *S* wave picks. Event magnitudes were based on solutions provided by INVOLCAN.

Tomographic inversion was based on the local earthquake tomography code LOTOS^20^, which has been used to investigate dozens of different volcanoes^10,21–23^. First, event locations in the reference 1D velocity model were determined using a grid-search method and linear approximation of ray paths^10^. We used the topography surface to limit the depths of events; therefore, events can be located above the sea surface. During this step, we selected events with eight or more picks and removed all data with absolute residual values of > 0.5 s for *P* waves and 0.7 for *S* waves. After removing outliers, the dataset used for the tomographic procedure included 11,349 events with 121,572 *P* wave and 127,766 *S* wave arrival times (mean of 22 picks per event).

Next, we relocated event sources using the gradient method and 3D bending algorithm for ray tracing^10^. In the first iteration, the relocation was conducted in the starting 1D model; in subsequent iterations, calculations were performed in the updated 3D model.

The 3D distributions of *P* and *S* wave velocity anomalies were parameterised using grid nodes irregularly distributed in the study area according to the ray density. Between the nodes, velocities were approximated continuously using bi-linear interpolation. The minimum grid spacing in both the horizontal and vertical directions was 0.7 km, which is considerably smaller than the size of resolved anomalies in our model. To reduce the influence of parameterisation on the results, we performed inversions in four grids with different basic orientations to the azimuthal direction (0, 22, 45 and 67 degrees) and then averaged them into one regular grid.

The inversion was performed simultaneously for the *P* and *S* velocity anomalies, source parameters (corrections of coordinates *dx, dy, dz*, and origin time *dt*), and station corrections. To stabilise the solutions, we used two types of regularisation—amplitude damping and flattening—which were performed by adding the corresponding equations to the general system. The values of the damping coefficients were determined from a series of synthetic tests with realistic anomaly sizes and noise levels. The derived sparse matrix was inverted using the Least Squares with QR-factorization (LSQR) method^24,25^.

The optimal 1D reference velocity model was derived after several runs of the complete tomographic procedure. After each trial, we calculated the average *P* and *S* velocities at certain depths and used them for the next iteration. As a result, we obtained a fair balance between high- and low-velocity anomalies at all depth layers.

After inversion in the four grids, the resulting *P* and *S* wave velocity anomalies were recalculated in a regular grid and then used for the next iteration, which included source relocation, matrix calculation, and inversion. In total, for the experimental and synthetic data, we performed five iterations, which was a compromise between solution stability and calculation velocity. The calculations enabled considerable variance reduction. In the L1 norm, the average *P* wave residuals reduced from 0.2397 to 0.0860 s (64.12%) and those for *S* waves reduced from 0.3058 to 0.1401 s (54.17%).

### Seismic tomography results

**Figure 3:**
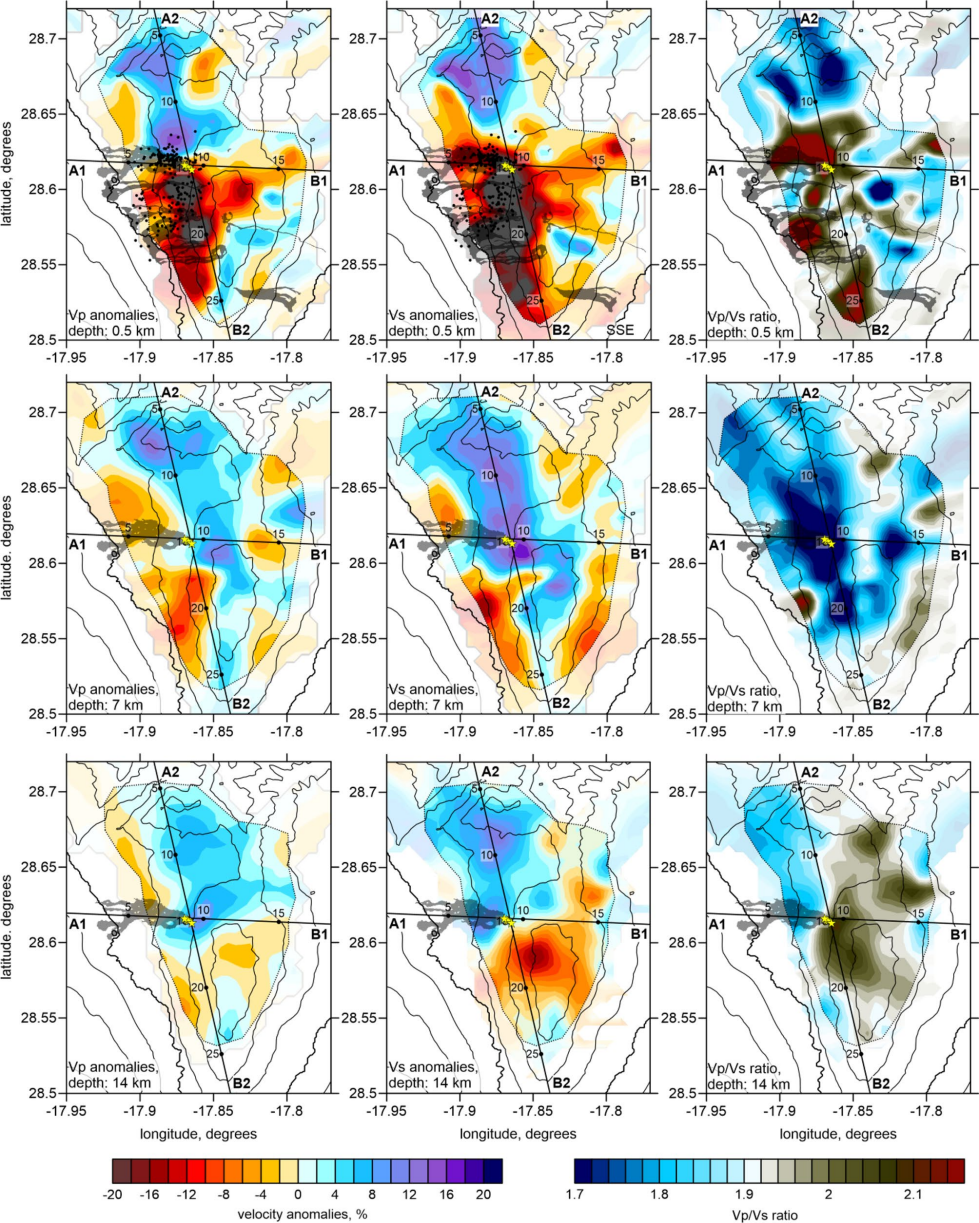
Horizontal layers representing P wave velocity anomalies (left column), S wave velocity anomalies (central column), and Vp/Vs ratio (right column) for three selected depths (0.5, 7 and 14 km depth b.s.l.). Black contour lines represent the topography with the interval of 500 m. In section at 0.5 km depth, all historical lava flows are shown; in other sections, only the flow of the 2021 eruption is presented. The yellow stars depict the vents of the 2021 eruption. The dotted line highlights the resolved areas based on the results of the checkerboard tests. The digital elevation model and historical lava flows were downloaded from the public graphic repository of GrafCan (www.grafcan.es). The 2021 lava flow was downloaded from the European agency Copernicus Emergency Management Service (httts://emergency.copernicus.eu/mapping/list-of-components/EMSR546). The software used to generate this figure was the LOTOS code.

**Figure 4:**
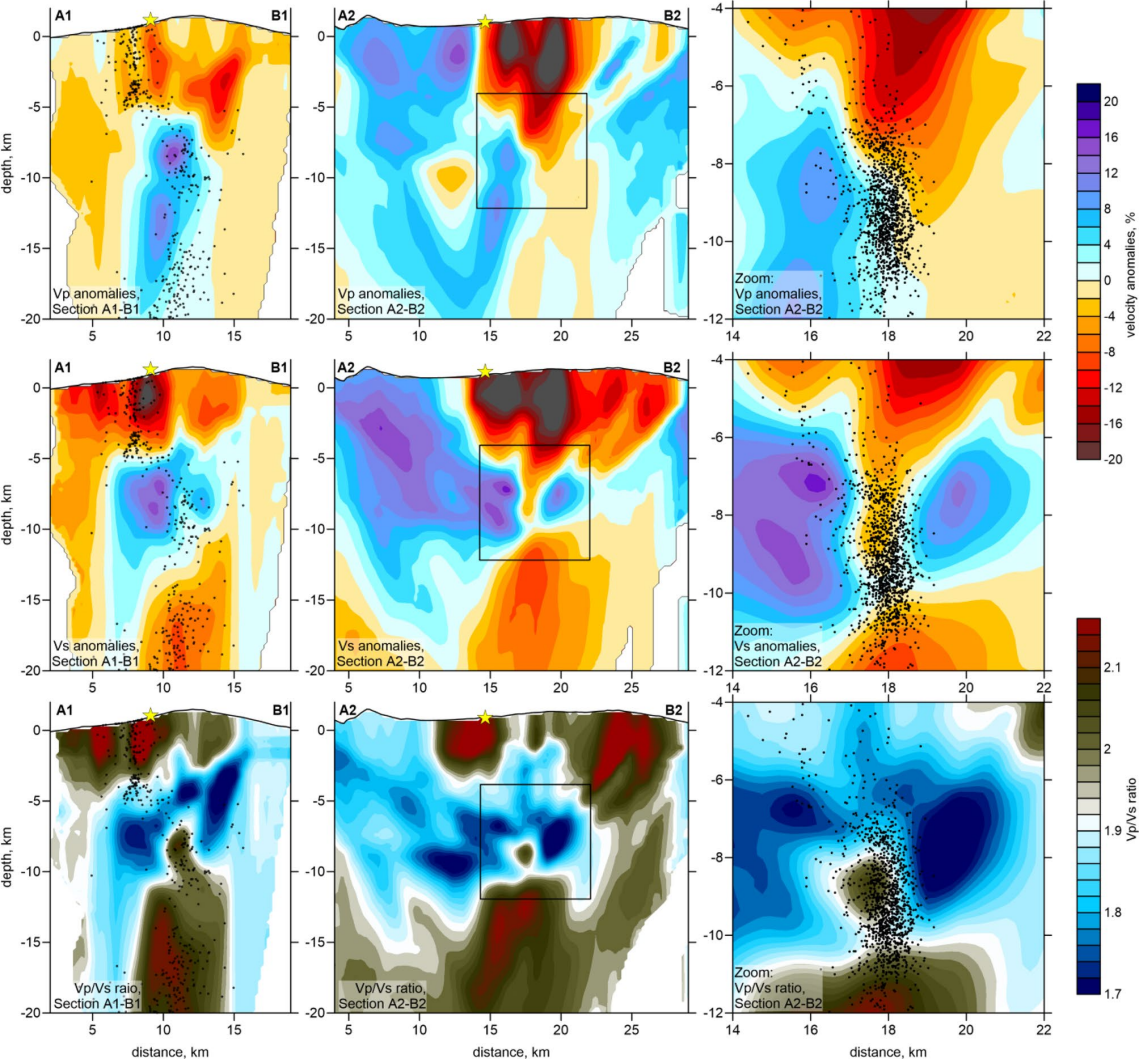
Vertical projections representing P wave velocity anomalies, S wave velocity anomalies, and Vp/Vs ratio. The locations of the profiles are shown in Fig. 3. The central squares of the A2-B2 profiles mark the area that is being zoomed in and is plotted in the right column. The black dots depict the event hypocenters located at distances of less than 0.6 km from the profile. The yellow star indicates the location of the 2021 eruption vent.

**Figure 5:**
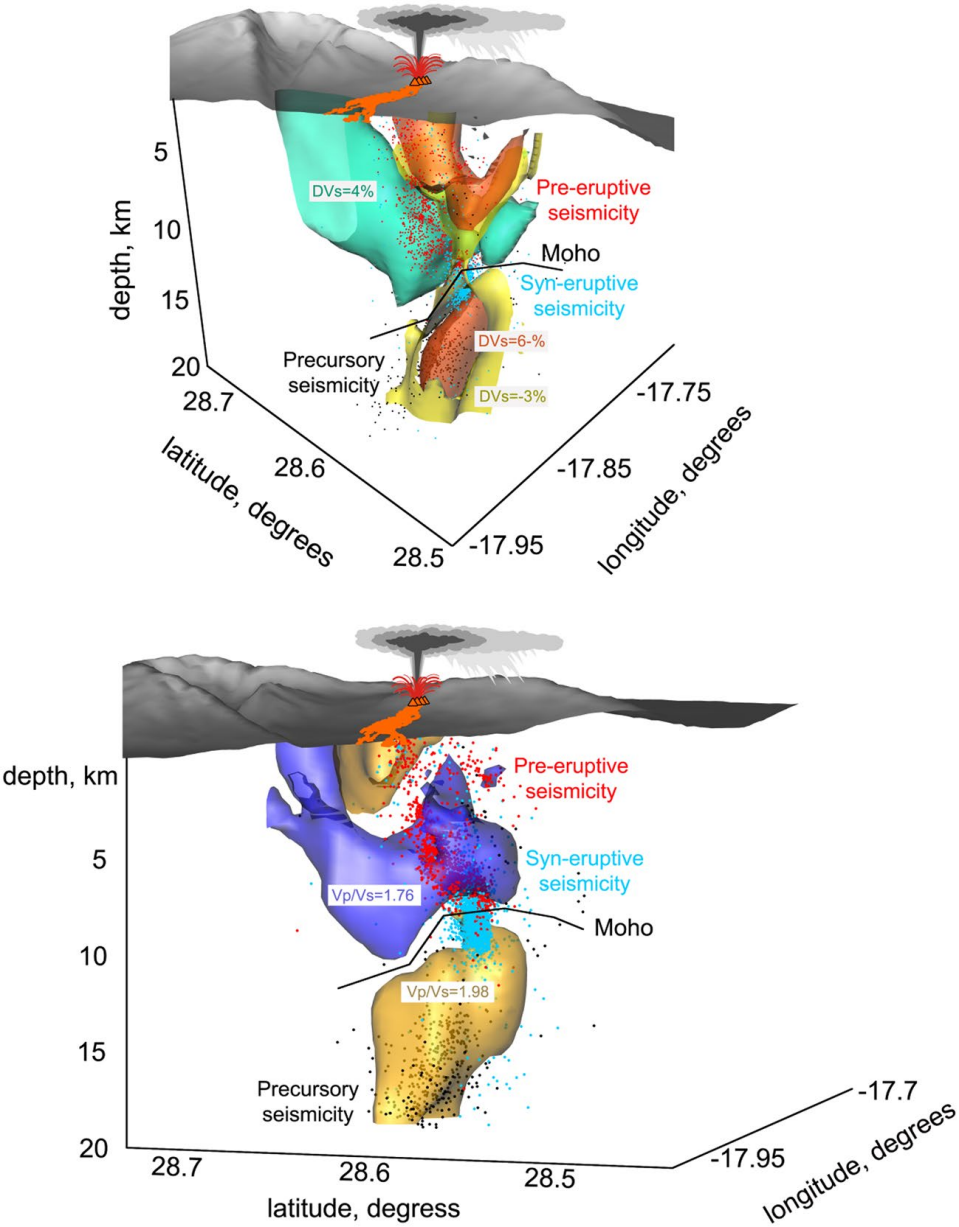
**Upper figure.** Three-dimensional image representing the main structures based on S waves velocity anomalies. Yellow and orange denotes bodies with lower velocity and green denotes bodies with higher velocity. The black line denotes the potential flexure of the Moho as a consequence of magma intrusion. Coloured dots denote the relocated seismicity according the occurrence phases. **Lower figure.** Same as Upper figure but based in the Vp/Vs ratio. Yellow bodies represent high Vp/Vs ratio and purple body represents low Vp/Vs ratio.

Figures 3, 4 and 5 show the inversion results of experimental data, including *P* wave velocity, and *S* wave velocity anomalies and *Vp/Vs* ratio in horizontal (Fig. 3) and vertical sections (Fig. 4), as well as in the 3D representation (Fig. 5). In the context of magma-related structures, it is important to present the *Vp/Vs* ratio, which was calculated by division of the derived *P* and *S* absolute velocities. The adequacy of this method was determined by the similar volumes of *P* and *S* wave data, and was confirmed using synthetic tests.

In the resulting tomography models, we observe highly heterogeneous structures with the deviations of velocities exceeding 20%. At shallow depths at 0.5 b.s.l. (or 1.5–2 km below surface in the central part of the island), we observe very strong low velocities of *P* and *S* waves beneath the southwestern slope of the island (upper row in Fig. 3). This anomaly almost perfectly matches with the locations of most vents of historical eruptions. It may also represent the presence of unconsolidated volcanoclastic deposits^26–29^. In the vertical sections (Fig. 4), we see that these low-velocity anomalies propagate down to ~ 5 km depth. The *Vp/Vs* ratio in shallow layers looks patchier but also exhibits a clear connection with the distributions of the vents. On the other hand, the area of the Taburiente Caldera (Fig. 1) in the northern part of the study area coincides with the areas of high velocities *Vp* and *Vs* and low *Vp/Vs* ratio. These findings are consistent with the results of magnetotelluric sounding^19^. An anomaly of positive dVp and dVs at the depth of 7 km depth in our results (Fig. 3, middle row) looks very consistent with the high-resistivity anomaly in the deepest Sect. (4–5 km) in Fig. 2 found by Di Paolo et al.^19^. At the shallower Sects. (0.5 km), the seismic anomalies are low in the south and high in the north (upper row in Fig. 3); nearly the same geometry is observed in the MT results at 1.996–2.379 km in which the areas of high resistivity were observed below Taburiente Caldera and low-resistivity anomalies were detected in the area of Cumbre Vieja vents.

At 7 km depth, we observe a prominent high-velocity and low *Vp/Vs* anomaly beneath the central part of the island. In vertical section A2-B2 in Fig. 4 and the 3D representation in Fig. 5, we see how this high-velocity layer is dissected by a vertically oriented low-velocity anomaly coinciding with a narrow seismicity cluster (see a zoomed fragment in the right column in Fig. 4). This seismicity probably represents the process of upward magma propagation through a new conduit formed due to fracturing of rocks in a brittle “blue” layer. In the lower part of this zone, we observe a “drop-shaped” anomaly of high *Vp/Vs* ratio that possibly indicates the ascent of partially molten magmas.

In the lower part of the model, below Cumbre Vieja, we observe a prominent anomaly of very high *Vp/Vs* ratio, which coincides with the distribution of deep seismicity (Fig. 5). Based on a similarity of this anomaly with structures observed beneath many other active volcanoes, we can conclude that it represents a deep conduit that delivers magma from deeper sources.

To assess the spatial resolution of the resulting models and to derive optimal values of controlling parameters for the tomographic procedure, we performed a series of synthetic tests. In all cases, the synthetic model was defined by a set of positive and negative anomalies with respect to the a priori 1D reference model. Synthetic travel times were calculated for the same source-receiver pairs as derived in the main experimental model after five iterations. The derived data were perturbed by random noise with an average deviation of 0.03 s for both *P* and *S* data, which enabled the same variance reduction as in the experimental data inversion. Before starting the synthetic model recovery, we “forgot” any information about the sources. Then, calculations were performed based on the same workflow as in the experimental data processing, including source location in the starting 1D model using the grid-search method. During synthetic modelling, we tuned the values of the controlling parameters to derive an optimal quality of the initial model recovery; then, the same controlling parameters were used for the experimental data inversion.

We separately investigated the resolution in the horizontal and vertical directions. In the first series of tests, we defined several checkerboard models with different anomaly sizes in map view. In Figure S4 of Supplementary, we present three tests with anomalies of 2, 3, and 4 km separated by 1 km spacing with zero anomaly values. In all cases, the amplitudes of anomalies were ± 8%. We defined the opposite signs of the *dVp* and *dVs* anomalies to enable contrasted variations of the *Vp/Vs* ratio. The recovery results are presented in two depth sections. Anomalies of 2 km in size were only resolved in the central part of the study area, where most earthquakes were located. For the models with larger anomalies, fair resolution was observed in most parts of the study area. Based on the results of these tests, we defined a contour of the resolved area, in which the results of experimental data were plotted (dotted line in Fig. 3). From these tests, we confirmed that the distribution of *Vp/Vs* was correctly recovered, demonstrating the adequacy of the method for this parameter calculation.

In another series of tests shown in Figure S5 of Supplementary, we explored the vertical resolution. Regarding the trade-off between velocity and source coordinates in passive source tomography, as well as dominantly vertical orientations of ray paths, we could expect poorer vertical resolution compared with horizontal resolution. In particular, there was a concern about the capacity of the existing data to provide abrupt changes in velocity at ~ 10 km depth, as observed in the experimental data inversion. To address this problem, we defined models with alternated anomalies defined in each of the vertical sections, in which the main results are presented. Along these sections, the anomalies had a size of 4 km and a spacing of 2 km. In the vertical direction, they formed two rows with an interval of zero values at depths between 6 and 10 km. This test confirmed that major anomalies in the central part of the study area were correctly recovered; however, some diagonal smearing is observed in marginal regions. This effect was taken into account during interpretation.

**Figure 6:**
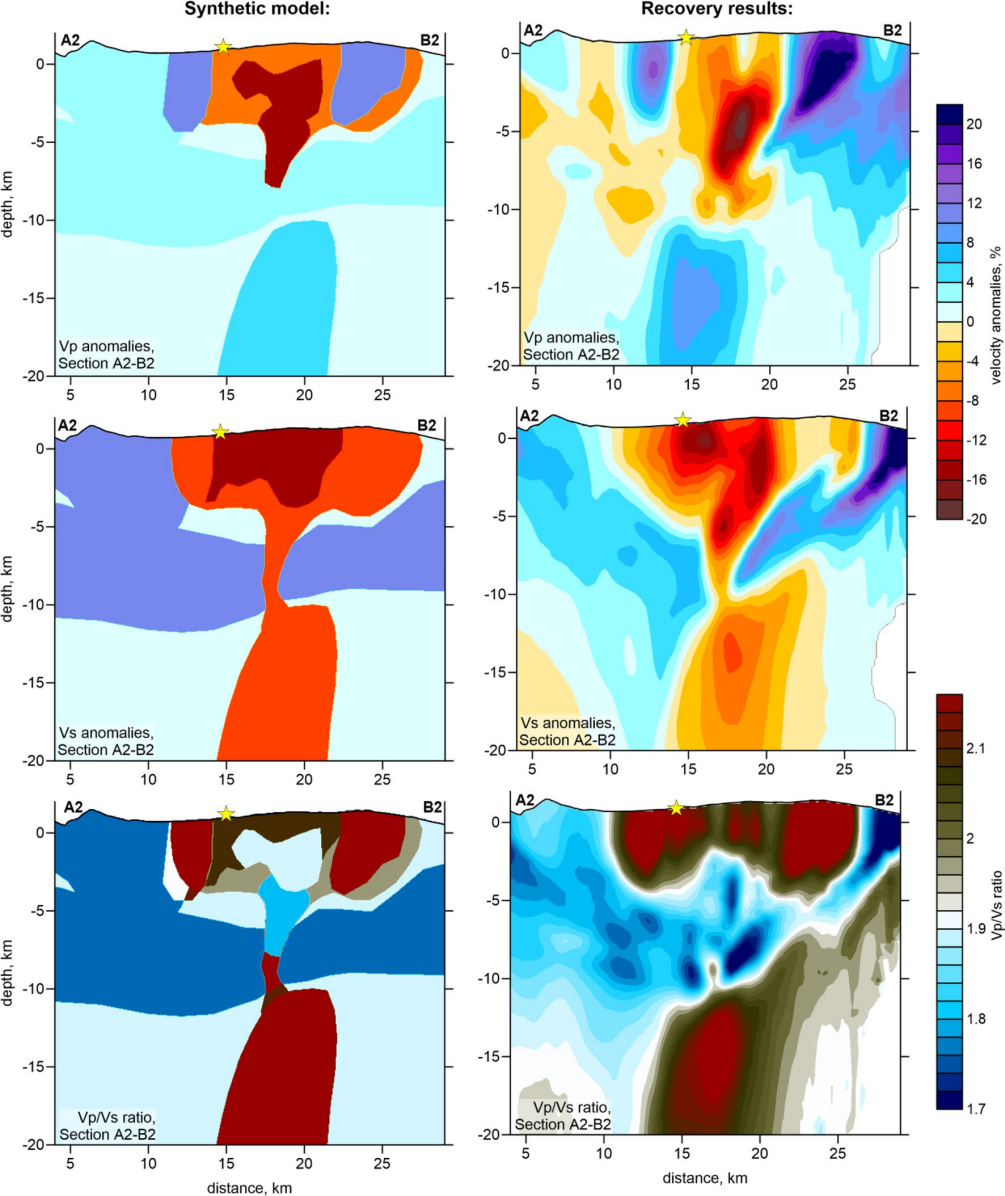
Resolution test with the synthetic model representing the realistic distributions of seismic structures along the section A2–B2. The configuration of the initial synthetic patterns of Vp, Vs anomalies and Vp/Vs ratio are presented in the left column, and the recovery results are shown in the right column. The yellow star indicates the location of the 2021 eruption vent.

The synthetic test with realistic anomalies shown in Fig. 6 demonstrates the capacity of the tomography inversion to recover the structures observed in the main model derived from the inversion of experimental data. The synthetic model in this case was defined by a set of polygons distributed along the vertical Section A2-B2. The anomalies of the *P* and *S* wave velocities were defined to enable the distributions of the recovered *dVp*, *dVs* and *Vp/Vs* ratio similar to those in the main model in Fig. 4. We see that all structures, which will be used for interpretation of the results in the Discussion section, could be robustly recovered in the case of this synthetic test.

As true event coordinates were presumed to be unknown in the recovery procedure, the synthetic tests allowed us to assess the accuracy of source locations. Figure S6 of Supplementary shows the mislocations of events in the model with a vertical checkerboard in Sect. 2 with respect to the true locations. When using the starting 1D velocity model, the average error of source coordinate determination in the L1 norm was 0.68 km. In the final model, the average error reduced to 0.42 km. As expected, the maximum errors were observed for events on the periphery of the network and at the greatest depths. These errors do not significantly affect the interpretation of the results presented in this manuscript.

## Discussion

**Figure 7:**
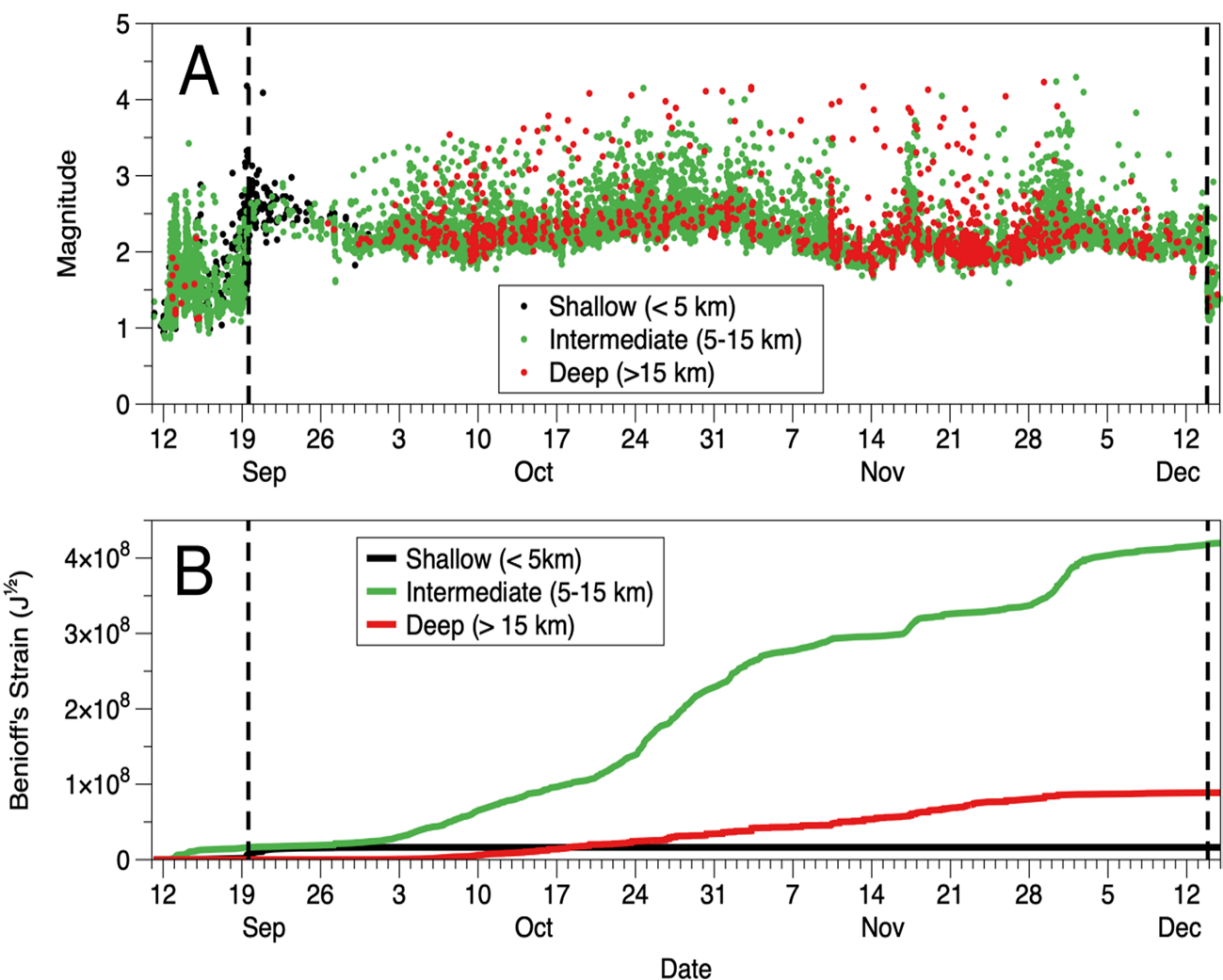
Earthquake magnitudes and Benioff’s strain release. (**A**) Magnitudes (Ml) of located earthquakes separated by depth; (**B**) accumulated Benioff’s strain release for each depth class. The dashed lines limit the active phase of the eruption.

Based on accurate relocations of the seismicity and magnitude determinations at different stages of the eruption development, we have estimated the earthquake Benioff’s strain release^29^ in selected earthquake clusters (Fig. 7). We found that the released seismic strain of intermediate seismicity (5–15 km) is almost four times larger than that of deep and shallow seismicity, suggesting that stress generation in this region is dominating the eruptive process. One of the most important characteristics of this seismicity is the difference between pre-eruptive and syn-eruptive seismicity. The pre-eruptive seismicity is of low magnitude and shows evident migrations from the deep towards the surface (Fig. 2, S1 and S2). The syn-eruptive seismicity is of much larger magnitude and presents two focal depth clusters, 10–12 km depth and 20–25 km depth with no apparent migration (Fig. 2 and S3). Note that the upper cluster roughly corresponds to the depth where the pre-eruptive seismicity began on 11 September 2021.

Our seismic tomography results offer improved understanding of pre- and syn-eruptive activity, along with the possible future volcanic scenarios in La Palma Island. Based on the calculated seismic velocity model and the seismicity distribution, we can single out the following observations:

1. The western Canary Islands, including La Palma, are underlain by high-velocity oceanic crust. Beneath most islands, the Moho (i.e., the base of the oceanic crust) is at ~ 10–12 km below sea level (bsl); this depth is greater than that between and surrounding the islands and is the expected consequence of isostatic equilibrium^6,17,19,30^. However, unlike the neighbouring island of El Hierro, where a volcanic unrest was observed in 2012, there is no crustal thickening beneath La Palma. On the contrary, our tomographic images suggest crustal thinning, with a high-velocity body extending from ~ 8 to 10 km bsl.
2. Underlying the volcanically active sector of La Palma is a spatially limited by an anomaly with high *Vp/Vs* > *2* and low velocities with negative *dVp* and *dVs* of more than 10% extending from the surface to ~ 3 km bsl. This structure has previously been identified as a low-resistivity hydrothermal zone^19^.
3. The third structure, characterised by high *Vp*, very low *Vs* with the magnitude of more than 10%, and high *Vp/Vs* > 2.05, extends between 7 and 25 km b.s.l. beneath the volcanically active sector of La Palma and represents partially molten material pooled at the base of oceanic crust. Based on our tomographic images, we estimate the volume of this magma-filled rock volume to be around 400 km^3^, dwarfing the other structures resolved by tomography. The calculation is a conservative estimate based on a simple geometric approximation of the high *Vp/Vs* ratio beneath the Moho. We have not considered the entire low velocity region for this estimate, but only the area covered by iso-lines larger than 1.98, as represented in Fig. 5b. In addition, the upward bending of the Moho seems to indicate that the formation of magma chambers in this same position occurred repeatedly during geological times, leading to a permanent modification of the Moho shape.

**Figure 8:**
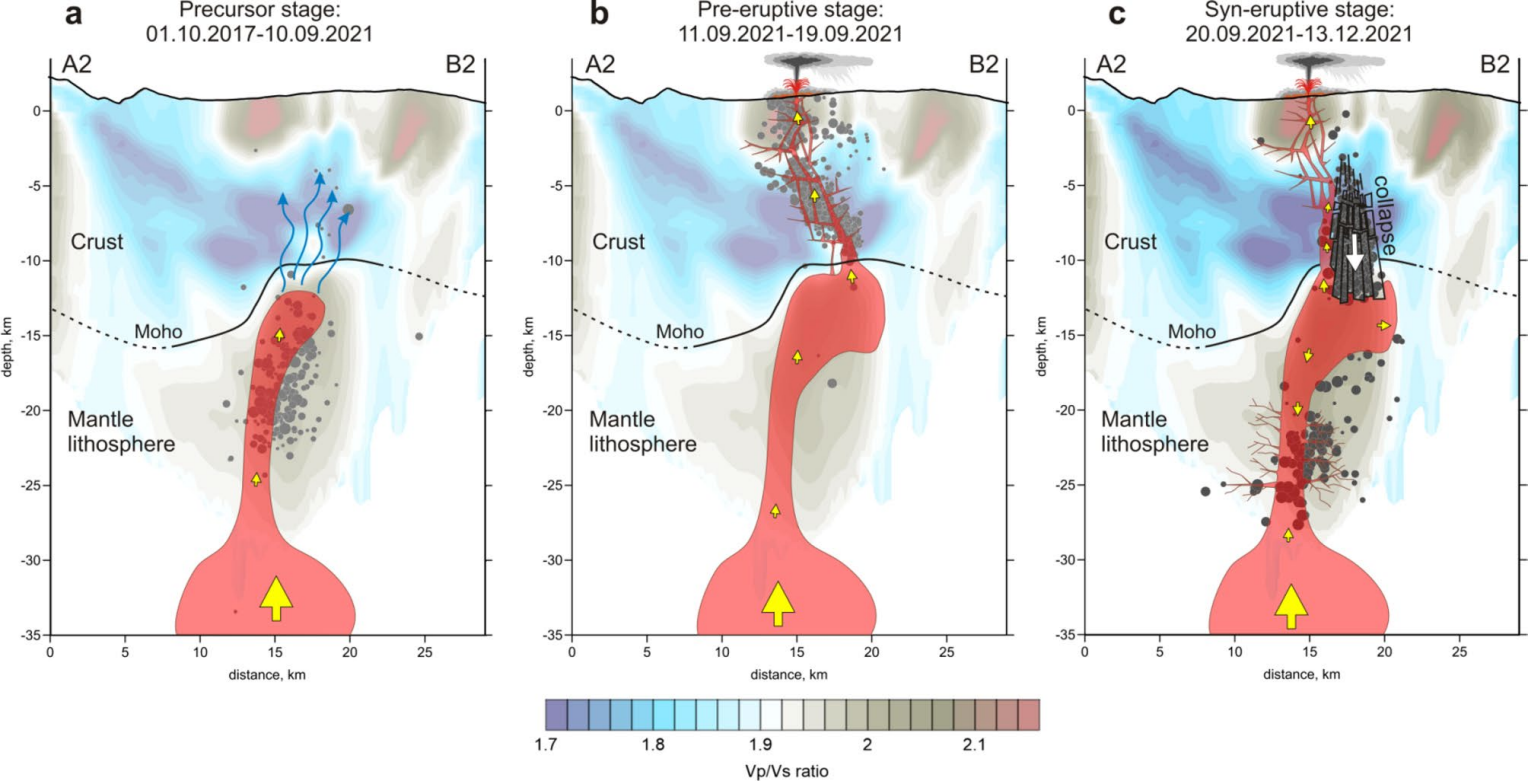
A sketch of the eruptive process preceding and accompanying the 2021 La Palma eruption. Background is the distribution of the Vp/Vs ratio in vertical section A2–B2 in the SSE-NNW direction, same as shown in Fig. 3. Dots are the projections of the event hypocenters in the corresponding time intervals at distances of less than 0.6 km; their size represent the magnitude. (**a**) Precursor stage. (**b**) Pre-eruptive stage. (**c**) Syn-eruptive stage. More description is presented in the main paper.

Based on these findings, we developed a scenario of the ongoing volcanic activity on La Palma, which is schematically demonstrated in Fig. 8.

1. For ~ 2 million years, repeated accumulations of magma^31^ have deformed the base of the oceanic crust beneath La Palma, as evidenced by upward displacement of the Moho and the shape of tomographic structures. Between October 2017 and August 2021, at least 9 earthquake swarms (producing ~ 700 well-located earthquakes) recorded magma injections from the mantle to the base of the oceanic crust beneath Cumbre Vieja volcano (the locus of all historical eruptions, including the present activity), causing the accumulation of a large magma-filled rock volume (Fig. 8a). This process, although likely continuous, did not cause significant stress changes, as evidenced by the sporadic and low-magnitude seismicity (Ml < 2.0). Initial earthquakes delineate the upper limit of the reservoir (i.e., the Moho or lower limit of consolidated oceanic crust). The foci of later precursory events trace fluid migration towards the surface and the exchange of stress with consolidated oceanic crust in the months before the eruption (Figs. 8a and S1 of Supplementary).
2. During the precursory phase (October 2017—August 2021), the seismicity was mostly grouped into short-lived seismic swarms (Fig. 2). Earthquake hypocentres were mostly located in the depth range 10–25 km and magnitudes were generally lower than 2. After a month of seismic quiescence, in the 7 days before eruption onset (approximately at 14:00 UTC on 19 September), we observe rapid upward migration of the pre-eruptive seismicity indicating magma ascent from 10 km bsl (i.e., the base of the Moho) to the surface along a zone of structural weakness delineated by a low velocity tomographic anomaly associated with seismicity that occurred a few months earlier (Figs. 8b and S2 of Supplementary). For the first 3 km, this low-velocity anomaly is vertical, but close to the surface it follows a NNW–SSE trend and dips ~ 30 degrees towards the current eruptive centre. The migration of seismicity in the shallow crust follows the boundary between consolidated oceanic crust and the hydrothermally altered zone. Despite the highly fractured and brittle nature of the hydrothermal zone, which theoretically offers low resistance to magma ascent, we hypothesise that the contact zone must present even lower mechanical resistance, which is consistent with the small magnitudes of earthquakes in a few days before the eruption that started to increase only a few hours prior to the opening of the vent (Fig. 7a). Similar associations of active volcanic vents with the contact zones between high and low seismic velocities are observed in many cases, such as Mount Saint Helens^32^, Tolbachik^33^ and Colima^34^, among other examples.
3. Seismicity during the first few days of eruption was characterised by energetic volcanic tremor (Figure S7). However, on 29 September, 10 days after eruption onset, an intense seismic swarm (in which most events had magnitudes of Ml > 3.0) occurred along the contact between the magmatic body and lower limit of the oceanic crust (~ 10 km). This seismicity, which is ongoing just until reaching the current period of eruptive calm on December 13, 2021, can be explained by the collapse of brittle crustal material above the reservoir owing to the continuous extraction of magma to the surface as schematically shown in Fig. 8c. In addition, since early October, a new earthquake cluster (Ml > 3.0) has been observed at ~ 17–30 km depth (Fig. 2d). This deeper seismicity, which shows no preferential lineation or temporal migration, includes the highest earthquake magnitudes recorded during the eruption (up to Ml = 4.3) and is located above a deep upper-mantle magma reservoir (close to the lithosphere base), as reported by previous studies^35,36^. We attribute this cluster to two possible explanations.
4. Magma compression caused by the collapse of crustal material had a piston effect; increased pressure at 12–15 km pushed magma both upwards and downwards (Fig. 8c). The opposing force of magma ascending from the mantle created excessive pressure in the conduit between the upper and lower reservoirs, which triggered fracturing of rocks around the conduit. We do not know any case where exactly the same mechanism occurred. In some way, it looks similar to the case of Katmai-Novarupta, where the collapse of the crater in Katmai pushed the magma in the reservoir and triggered a strong explosive eruption of Novarupta in June 1912^37^. The existing estimates of focal mechanisms of events during this period show very complex and variable patterns that might take place in the case of an internal collapse presuming the existence of all types of displacements.
5. Alternatively, by invoking stress propagation model similar to that of the 2011–2014 eruption of neighbouring El Hierro^38^, this activity could record the collapse of magmatic reservoir owing to magma withdrawal and corresponding depressurization. It is interesting to note that earthquakes hypocentres during the precursory phase were mostly located within the depth range delimited by the two syn-eruptive clusters. This suggests that precursory seismicity was related to magma-transfer episodes which led to a progressive filling of the intermediate magma chamber, until the onset of the eruption. During the eruption the only significant occurrence of earthquakes within this depth range was observed at the end of November (Fig. 2).

## Conclusions

Our tomographic images mark a milestone in the field of volcano seismology, and provide valuable insight into the short-term evolution of a magma plumbing system from the upper mantle to the surface. In particular, given the large size of the magma reservoir that feeds the volcanic eruption it is not possible to discard this magmatic system could cause future new volcanic eruptions on the Island of La Palma.

Among the lessons learned, of relevance for volcano monitoring are:

1. The pre-eruptive stage can be faster than expected. In the case of Cumbre Vieja 2021 eruption, it was about 7 days. Furthermore, we observe the rapid ascent of the hypocenters in the very few hours preceding the eruption (Fig. 2). Therefore, we conclude that the decision-making process during a volcanic emergency should not rely on more or less constant trends: sudden changes can occur any time.
2. The 3D relocation of hypocenters, is fundamental for understanding precisely the evolution of the seismicity and therefore the dynamics of the magmatic system in near-real-time. A tomographic model, if not already existing, can be obtained quickly, just few days after the onset of a volcanic unrest, if a sufficient number (> 500) of earthquakes is available.
3. Seismic tomography can identify crustal structure relevant for the propagation of magmatic intrusions. Hence, it provides a valuable tool for volcanic hazard studies.

## Supplementary Information

Supplementary Information.

## Data Availability

The seismic catalogue of IGN is publicly available at: https://www.ign.es/web/ign/portal/sis-catalogo-terremotos. The seismic catalogue of INVOLCAN is available under request to Dr. Luca D’Auria (ldauria@iter.es). The LOTOS code is publicly available at: www.ivan-art.com/science/LOTOS. An online version of the code with the La Palma dataset is available in: Koulakov Ivan. (2022). Data and program codes to reproduce the results of seismic tomography for La Palma Island [Data set]. Zenodo. 10.5281/zenodo.6589367. The digital elevation model used in all figures and historical lava flows of Figs. 1 and 3 were downloaded from the public graphic repository of GrafCan (www.grafcan.es). The 2021 lava flow was downloaded from the European agency Copernicus Emergency Management Service (httts://emergency.copernicus.eu/mapping/list-of-components/EMSR546). The software used to generate Fig. 1, Figs. S1, S2 and S3 was QGIS 3.22 (https://www.qgis.org). The software used to generate Figs. 3, 4 and  6, Figs. S4, S5 and S6 is the LOTOS code.
