# Supplementary material for: Rapid magma ascent beneath La Palma revealed by seismic tomography
Source: Sci Rep. 2022 Oct 21;12:17654. doi: 10.1038/s41598-022-21818-9 (PMC9587211; doi:10.1038/s41598-022-21818-9)
Supplement: Supplementary file 1 — Supplementary Information. [file 41598_2022_21818_MOESM1_ESM.docx]

**Rapid magma ascent beneath La Palma revealed by seismic tomography**

Luca D'Auria^1,2^, Ivan Koulakov^3,4,5^, Janire Prudencio^6,7^, Iván Cabrera-Pérez^1^, Jesús M. Ibáñez^6,7 *^, Jose Barrancos^1,2^, Rubén García-Hernández^1^, David Martínez van Dorth^1,2^, Germán D. Padilla^1,2^, Monika Przeor^1,2^, Victor Ortega^1^, Pedro Hernández^1,2^, Nemesio M. Peréz^1,2^

1. Instituto Volcanológico de Canarias (INVOLCAN), Calle Álvaro Martín Díaz, 2, San Cristóbal de La Laguna, Tenerife, Spain
2. Instituto Tecnológico y de Energías Renovables (ITER), Polígono Industrial de Granadilla s/n, 38600, Granadilla de Abona, Tenerife, Spain
3. Trofimuk Institute of Petroleum Geology and Geophysics SB RAS, Prospekt Koptyuga, 3, 630090 Novosibirsk, Russia
4. Novosibirsk State University, Novosibirsk, Russia, Pirogova 2, 630090 Novosibirsk, Russia
5. Institute of the Earth’s Crust SB RAS, Lermontova 128, Irkutsk, Russia
6. Department of Theoretical Physics and Cosmos. Science Faculty. Avd. Fuenteneueva s/n. University of Granada. 18071. Granada. Spain.
7. Andalusian Institute of Geophysiscs. Campus de Cartuja. University of Granada. C/Profesor Clavera 12. 18071. Granada. Spain.

* Corresponding author: Jesús M. Ibáñez (jibanez@ugr.es)

**Extended data figures**


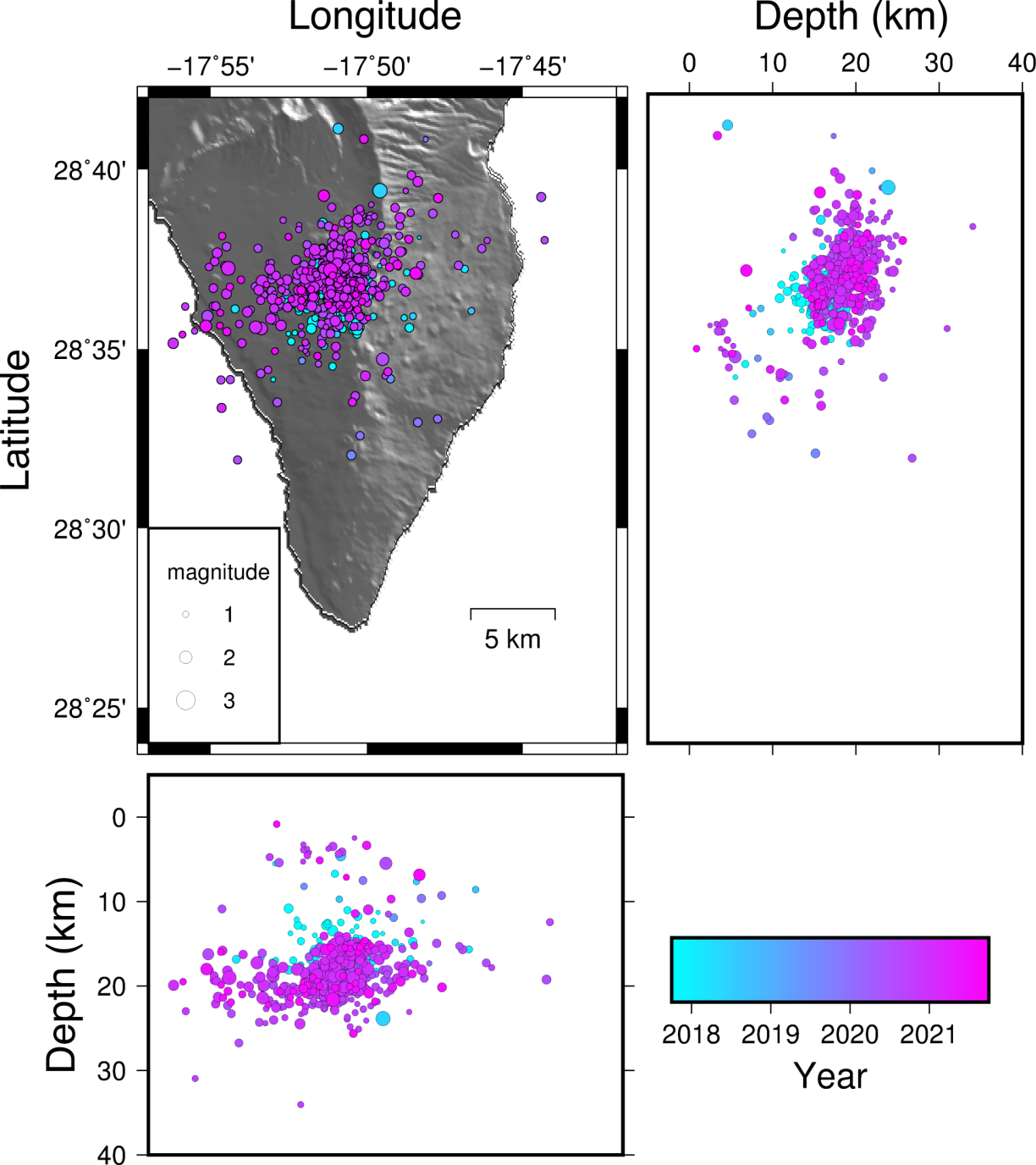


Figure S1. Spatio-temporal distribution of relocated precursory seismicity (From the end of 2017 to August 2021) showing the epicentral distribution and foci in N–S and E–W projections. The temporal scale is represented in terms of years.


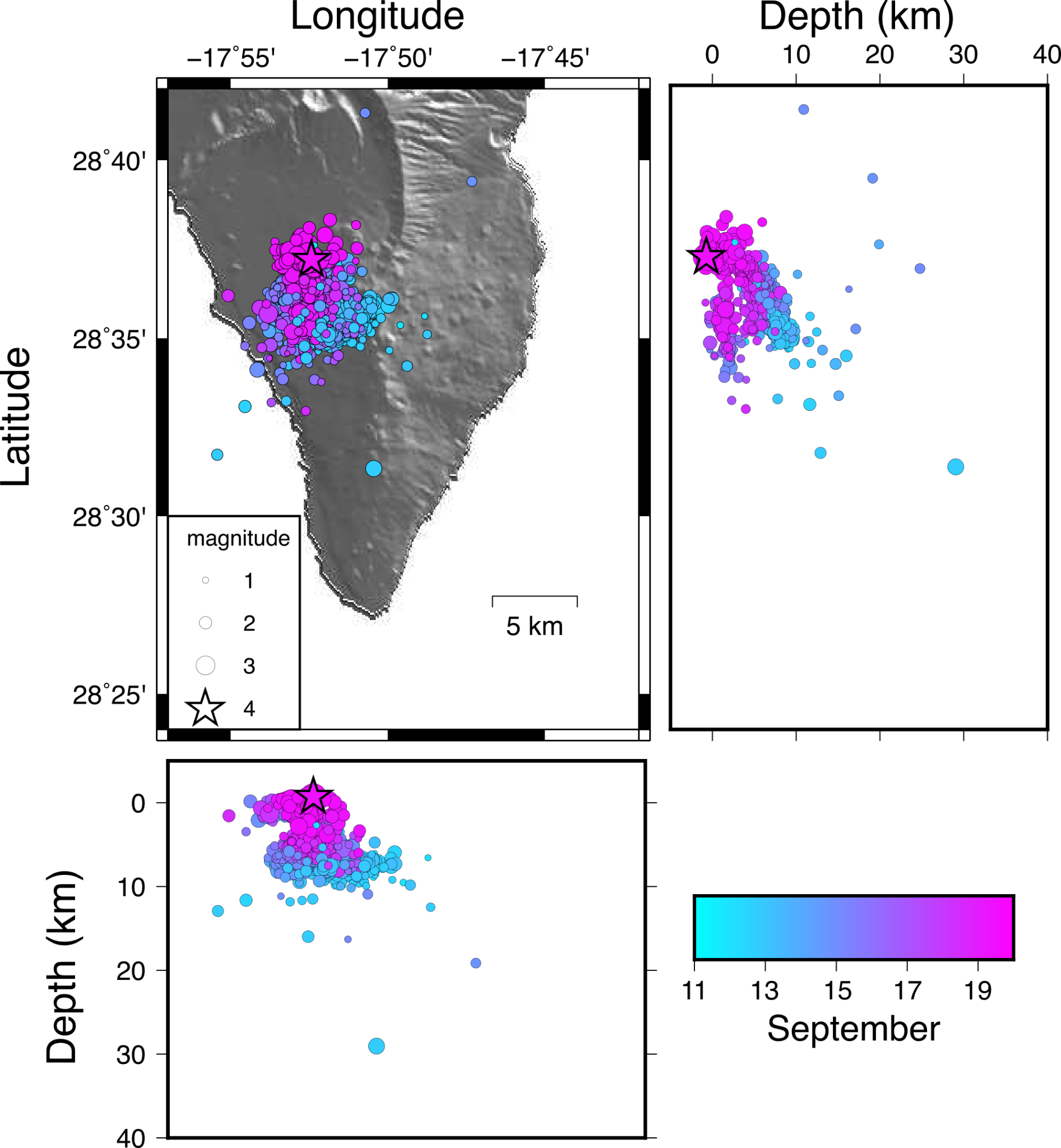


Figure S2. Spatio-temporal distribution of relocated pre-eruptive seismicity (from 11 to 19 of September 2021) showing the epicentral distribution and foci in N–S and E–W projections. The temporal scale indicates the days of September 2021.


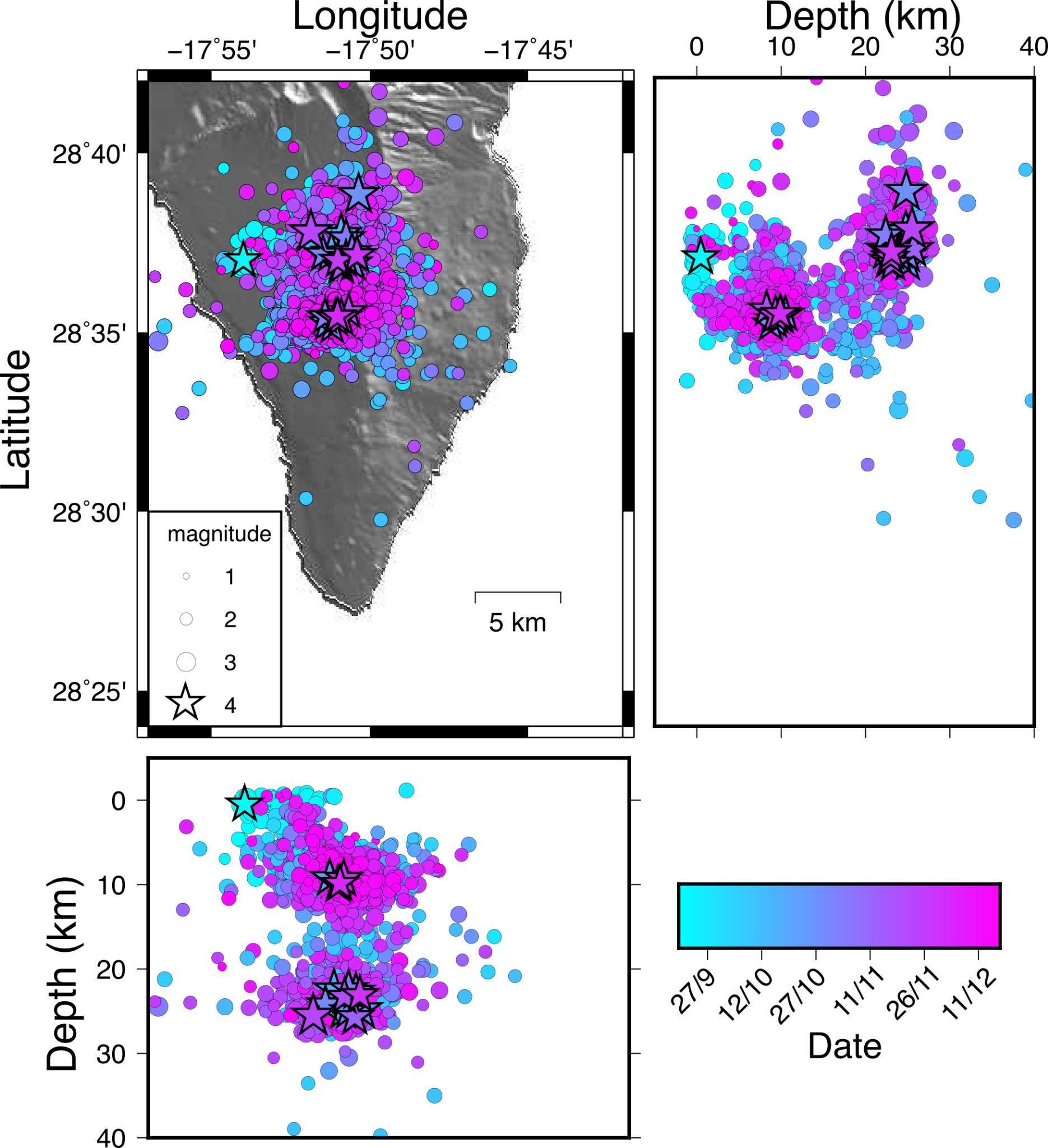


Figure S3. Spatio-temporal distribution of relocated syn-eruptive seismicity showing the epicentral distribution and foci in N–S and E–W projections. The temporal scale spans from 19 of September to 13 of December.

**
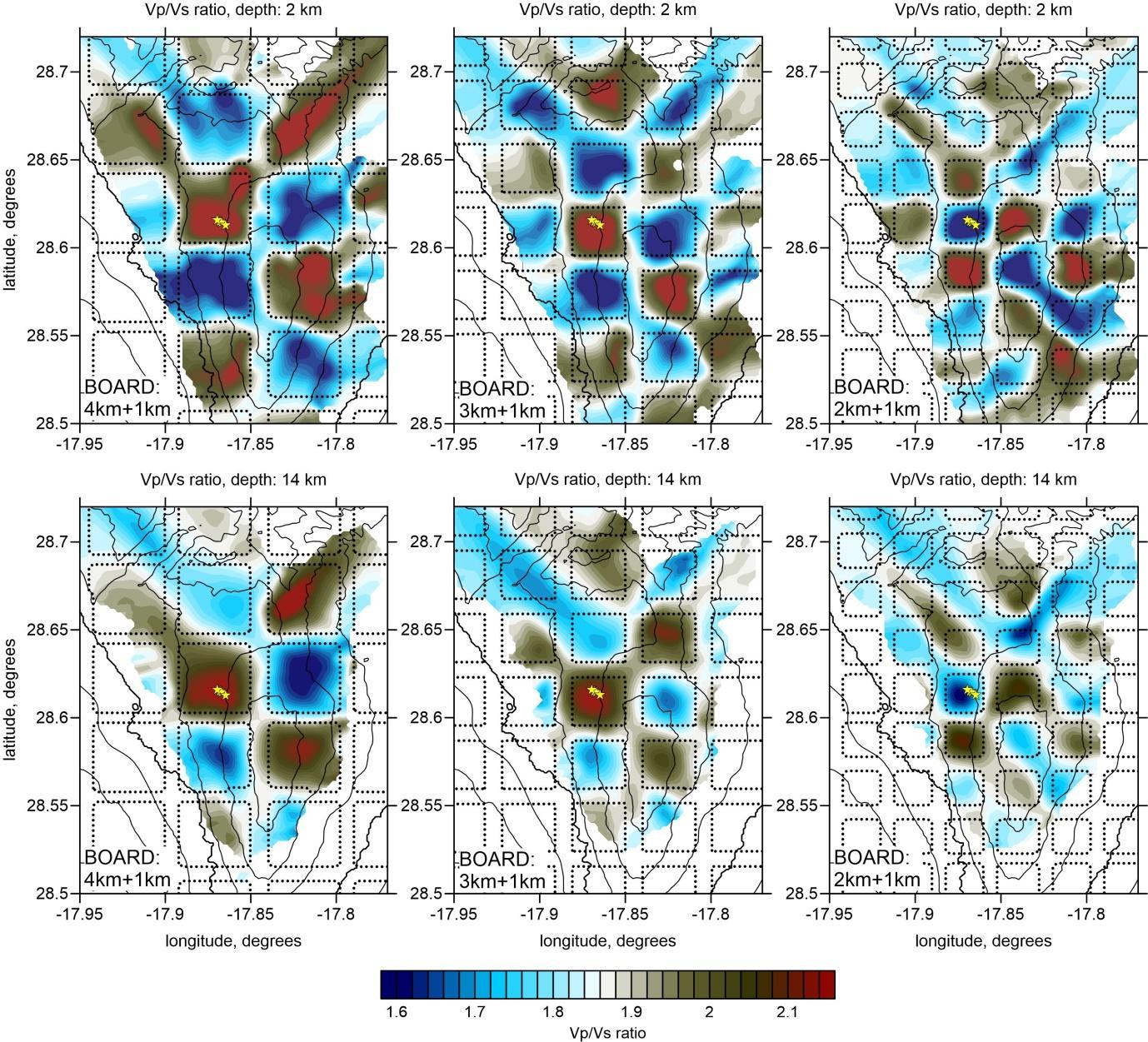
**

Figure S4. Resolution test results based on the inversion of experimental Vp/Vs data shown in horizontal layers. Black contour lines represent the topography with the interval of 500 m. The sizes of the synthetic anomalies and spacing are indicated in bottom-left corner.


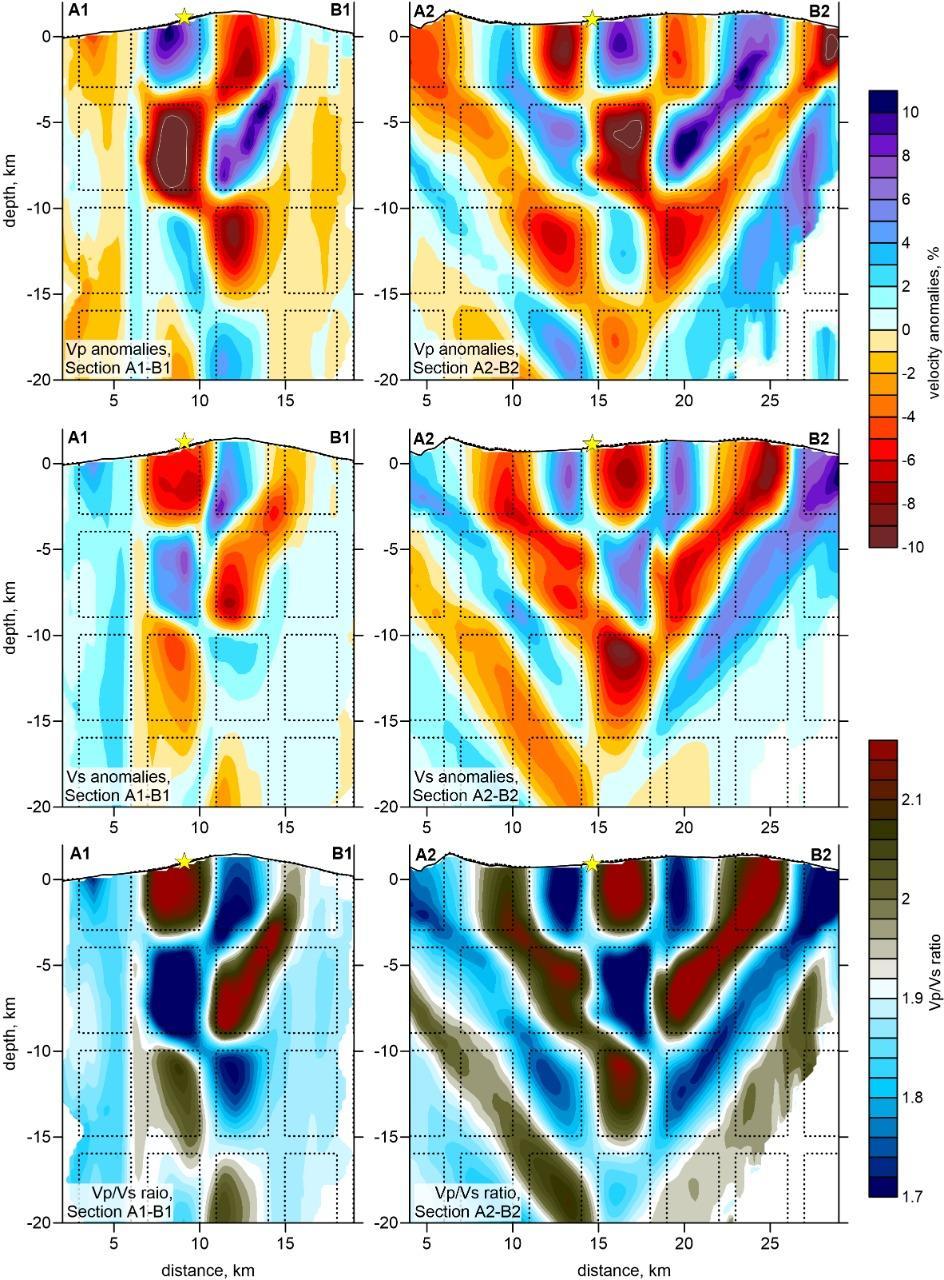


Figure S5. Resolution test results based on the inversion of experimental *Vp*, *Vs* and *Vp/Vs* data shown in vertical layers. The yellow star indicates the location of the 2021 eruption vent.


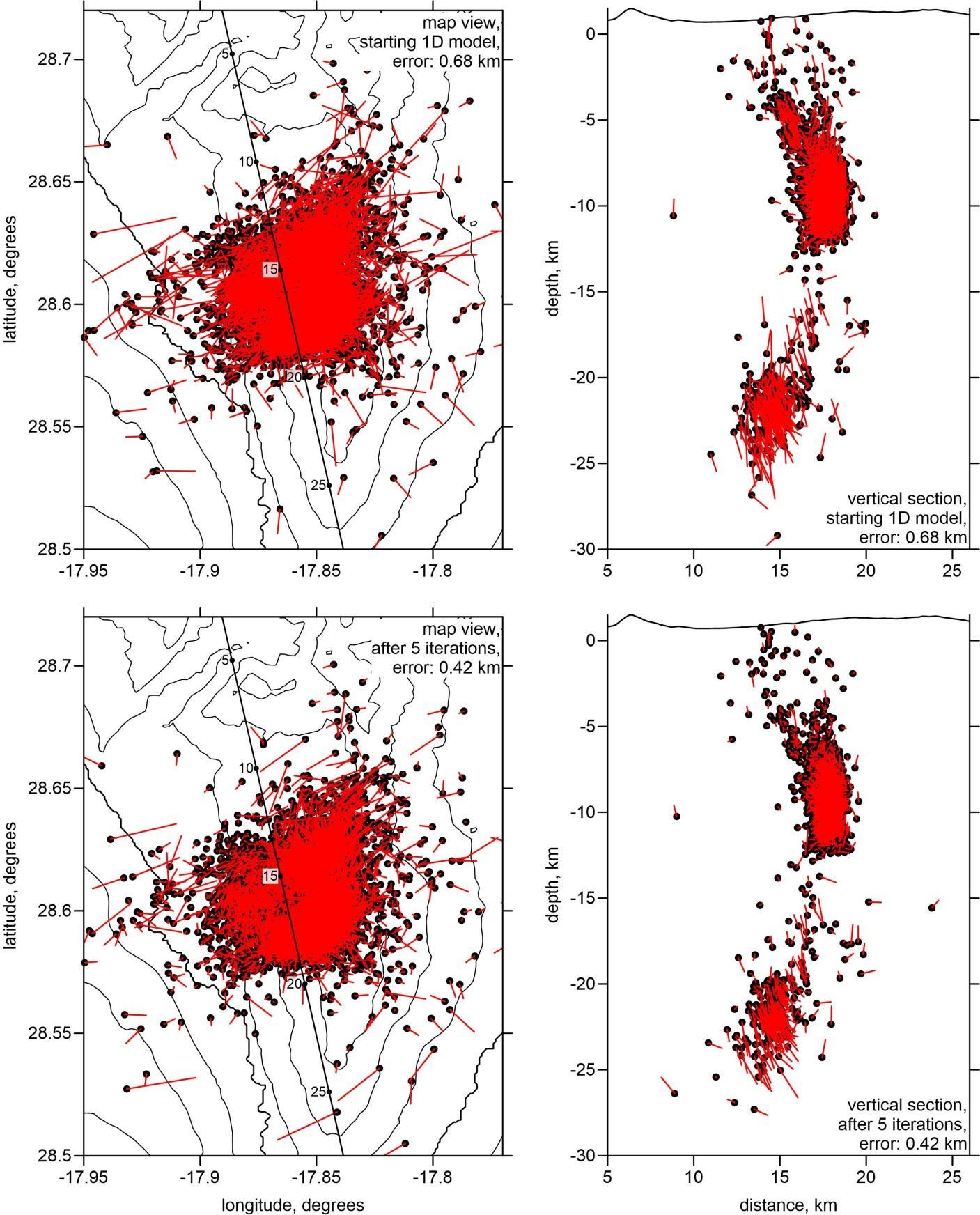


Figure S6. Event mislocations within the model with respect to true locations in horizontal and vertical views and with a vertical checkerboard in section 2. The black dots indicate the current locations; the red bars direct to the true locations. In profiles, the black line indicate the relief.


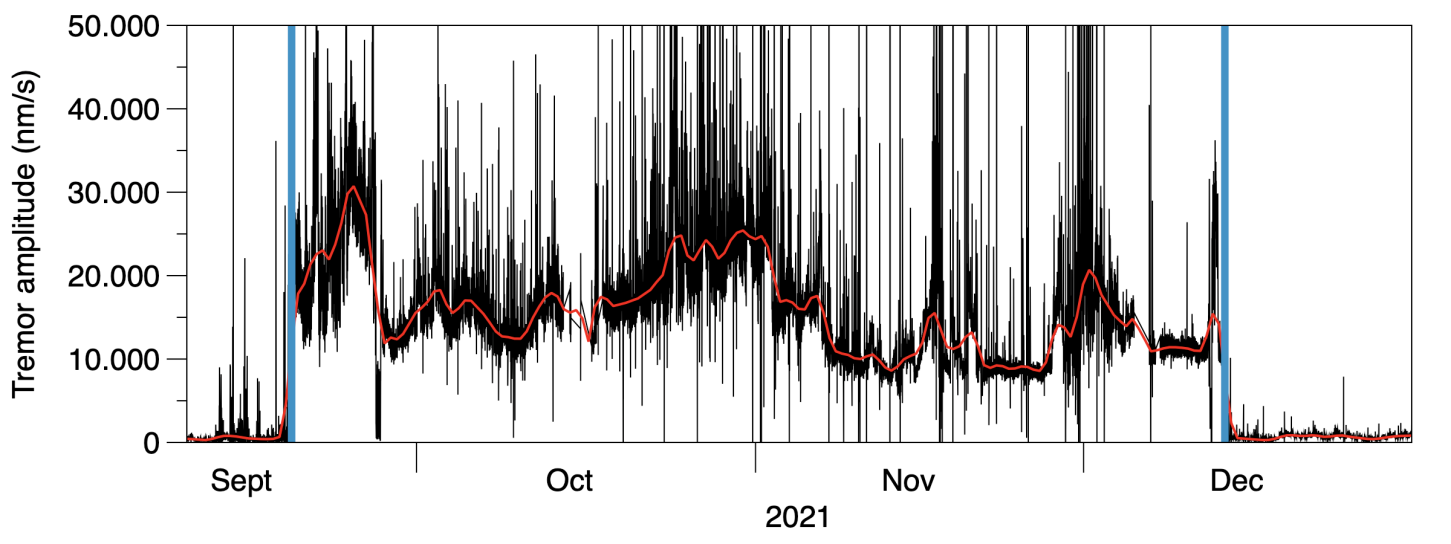


Figure S7. Volcanic tremor amplitude during the eruption. The graph shows the volcanic tremor amplitude recorded at station PLPI, located 4 km S of the eruptive centre. The amplitudes have been computed on the vertical component filtered in the band 1-5 Hz. The black curve represents the tremor amplitude averaged on one-minute windows, while the red curve represents the same value averaged on windows of one day. The blue vertical lines mark respectively the start and the end of the eruption. Peaks on the black curve are related to local earthquakes.
